# Supplementary material for: HIV and hepatitis B virus co-infection in Mozambique: Policy review and health professionals’ knowledge and practices
Source: PLoS One. 2024 Aug 20;19(8):e0301305. doi: 10.1371/journal.pone.0301305 (PMC11335122; doi:10.1371/journal.pone.0301305)
Supplement: S1 Table — (DOCX) [file pone.0301305.s001.docx]

S1 Table. Documents related to the existing HIV/Hepatitis B virus co-infection policies.

| **Nr** | **Name of document** | **Year** | **Issuing authority** | **Key information regarding policy:** | | |
| --- | --- | --- | --- | --- | --- | --- |
|  |  |  |  | Prevention | Diagnosis | Treatment |
| 1 | Portaria n° 325/77 | 1977 | MoH | Blood safety for transfusion policy | Blood testing for HIV, Syphilis HBV, HCV | No reference to Hepatitis B treatment |
| 2 | Decreto 14/88 | 1988 | MoH | Creates the National Transfusion Program | Not applicable | Not applicable |
| 3 | Lei 4/98 (Medicines Law) | 1998 | MoH | Not applicable | Not applicable | Regulates the availability of medicines |
| 4 | Diploma Ministerial n°183-A/2001 | 2001 | MoH | HIV post-exposure prophylaxis to health professionals | No reference to Hepatitis B | No reference to Hepatitis B |
| 5 | Plano Estratégico Nacional de Combate ao HIV/SIDA (PEN II) 2005-2009 | 2004 | MoH | Safe Transfusions and Injections  Hepatitis B Vaccine | Blood testing according to WHO recommendations | No reference to Hepatitis B |
| 6 | Formulário Nacional de Medicamentos | 2007 | MoH | Hepatitis B Vaccine | No reference to Hepatitis B | 3TC for the Treatment of HIV and HBV |
| 7 | Guia de tratamento antiretroviral e Infecções oportunistas no Adulto, Adolescente e Grávida | 2009 | MoH | Hepatitis B vaccine to Health professionals after exposure and to sexual assault victims | No reference to Hepatitis B diagnosis | Syndromic treatment of STIs in the HIV+ patient  It does not refer to the treatment of Hepatitis B |
| 8 | Blood Donation Screening Guide for detection of transfusion-transmissible infections | 2010 | WHO | Mandatory screening of all blood donations | Blood testing for HBV, HCV, HIV and Syphilis | Not applicable |
| 9 | Guia de tratamento antiretroviral e Infecções oportunistas no Adulto, Adolescente, Grávida e Criança | 2014 | MoH | Hepatitis B vaccine to Health professionals after exposure and sexual assault victims  Does not refer to Hepatitis B prevention in HIV+ patients | Hepatitis B testing of exposed HCWs and victims of sexual assault | It does not refer to the treatment of Hepatitis B |
| 10 | Comprehensive Multi-Year strategic Plan (cMYP)  2015 – 2019 | 2014 | MoH | Hepatitis B vaccine at < 1 year. It does not refer to infants, HIV+ and Health professionals | Not applicable | Not applicable |
| 11 | Decreto 46/2015 | 2015 | MoH | Creates the National Blood Service | Not applicable | Not applicable |
| 12 | Guidelines for the prevention, care and treatment of persons with chronic hepatitis B infection | 2015 | WOH | HBV vaccine to the newborn after delivery; prevention of vertical transmission of HBV by treating monoinfected pregnant women with TDF and with TDF,3TC, EFV in co-infected women;  HBV vaccine to key populations and Health professionals | HBsAg and anti-HBs testing for all HIV+ patients | Treatment with TDF/3TC or TDF/Emtricetabine |
| 13 | Guia de tratamento antiretroviral e Infecções oportunistas no Adulto, Adolescente, Grávida e Criança | 2016 | MoH | Hepatitis B vaccine to Health professionals after exposure and sexual assault victims. | HBV testing of exposed HCWs and victims of sexual assault.  HIV+ patient testing is not mentioned | Treatment with TDF+3TC+EFV to all co-infected patients |
| 14 | Diploma Ministerial n°58/2016 | 2016 | MoH | Not applicable | Not applicable | Approves the List of Essential Medicines |
| 15 | Lei 12/2017 (Law on Medicines, Vaccines, Biological and Health Products for Human Use) | 2017 | MoH | Not applicable | Not applicable | Importation of medicines, vaccines and diagnostic tests |
| 16 | Lista de Medicamentos essenciais | 2017 | MoH | Not applicable | Not applicable | List includes HBV drugs |
| 17 | Despacho Ministerial | 2017 | MoH | Not applicable | Not applicable | Establishes the norms for importation of medical articles |
| 18 | Global Hepatitis report | 2017 | WHO | Vaccinations for < 1 year and Newborns  Safe injections and transfusions  Risk reduction in intravenous drug users | HBV testing of newly diagnosed HIV+ persons | Treatment of hepatitis B to those eligible for life |
| 19 | Circular “Registo Colaborativo de Medicamentos e Vacinas” | 2018 | MoH | Not applicable | Not applicable | Registration of HIV/AIDS, TB, Malaria, Hepatitis drugs |
| 20 | Resolução n°15/2018 | 2018 | MoH | Approves the Organic Statute of the National Blood Service | Not applicable | Not applicable |
| 21 | Consolidated Strategic  Information Guidelines for viral Hepatitis. Planning and tracking progress towards elimination | 2019 | WHO | Vaccinations for Children Under 1 Year Old and Newborn  Safe injections and transfusions  Risk reduction in intravenous drug users | HBV testing of newly diagnosed HIV+ persons | HBV treatment for those eligible for life |

HBV-Hepatitis B virus, HCV-Hepatitis C virus, HIV- human immunodeficiency virus, AIDS- acquired immune deficiency syndrome, STIs- sexually transmitted infections, TB-tuberculosis, HCW-Health Care Workers, TDF-Tenofovir, 3TC-Lamivudine, EFV-Efavirenz, HBsAg-Hepatitis B surface antigen, WHO- World Health Organization, MoH-Ministry of Health of Mozambique.
