# Supplementary material for: HIV and hepatitis B virus co-infection in Mozambique: Policy review and health professionals’ knowledge and practices
Source: PLoS One. 2024 Aug 20;19(8):e0301305. doi: 10.1371/journal.pone.0301305 (PMC11335122; doi:10.1371/journal.pone.0301305)
Supplement: S2 Table — (DOCX) [file pone.0301305.s002.docx]

S2.Table. Knowledge about co-infection and sociodemographic characteristics.

|  | **Do you know that HIV and HBV can exist in the same patient?** | | | | **Have you ever heard of HBV treatment in HIV positive patients?** | | | | **Do you know about HBV complications in patients with HIV?** | | | | |
| --- | --- | --- | --- | --- | --- | --- | --- | --- | --- | --- | --- | --- | --- |
|  | Yes  N (%) | No  N (%) | Total  N (%) | P | Yes  N (%) | No  N (%) | Total  N (%) | P | Yes  N (%) | No  N (%) | Total  N (%) | | P |
| **Gender** |  | | |  |  | | |  |  | | | |  |
| Female | 39 (78) | 1(50) | 40 (77) | ***0.412*** | 10 (83) | 30 (75) | 40 (77) | 0.433 | 9 (75) | 31 (78) | | 40 (77) | ***0.567*** |
| Male | 11(22) | 1(50) | 12 (23) |  | 2 (17) | 10 (25) | 12 (23) |  | 3 (25) | 9 (22) | | 12 (23) |  |
| **Profession** |  |  |  |  |  |  |  |  |  |  | |  |  |
| Doctor | 8 (16) | 0 (0) | 8 (15) | ***0.197*** | 5 (42) | 3 (12) | 8 (22) | ***0.009*** | 6 (50) | 2 (5) | | 8 (15) | ***0.001*** |
| Medical Technician | 9 (18) | 0 (0) | 9 (17) |  | 5 (42) | 4 (17) | 9 (25) |  | 1 (8,3) | 8 (20) | | 9 (17) |  |
| Nurse | 19 (38) | 0 (0) | 19 (37) |  | 2 (16) | 17 (71) | 19 (53) |  | 4 (33,3) | 15(37,5) | | 19 (37) |  |
| Counselor * |  |  |  |  |  |  |  |  | 1 (8,3) | 15(37,5) | | 16 (31) |  |
| **Professional Experience** |  |  |  |  |  |  |  |  |  |  | |  |  |
| 6 M-5 years | 21 (42) | 2(100) | 23 (44) | ***0.269*** | 4 (33) | 19 (48) | 23 (44) | ***0.345*** | 4 (33,3) | 19 (47) | | 23 (44) | ***0.568*** |
| 6 a 10 years | 17 (34) | 0 (0) | 17 (33) |  | 6 (50) | 11 (28) | 17 (33) |  | 4 (33,3) | 13 (32) | | 17 (33) |  |
| + 10 years | 12 (24) | 0 (0) | 12 (23) |  | 2 (17) | 10 (24) | 12 (23) |  | 4 (33,3) | 8 (20) | | 12 (23) |  |

**HIV-human immunodeficiency virus, HBV-hepatitis B virus**

***** **Counselors were excluded from this component as they do not treat patients.**
