# Supplementary material for: HIV and hepatitis B virus co-infection in Mozambique: Policy review and health professionals’ knowledge and practices
Source: PLoS One. 2024 Aug 20;19(8):e0301305. doi: 10.1371/journal.pone.0301305 (PMC11335122; doi:10.1371/journal.pone.0301305)
Supplement: S3 Table — (DOCX) [file pone.0301305.s003.docx]

S3 Table. Health Professionals Practices and sociodemographic characteristics

|  | **Do you screen for HBV in a patient with HIV?** | | | | | | **Do you counsel to prevent HBV transmission?** | | | | | | **Do you counsel on risky behavior?** | | | | | |
| --- | --- | --- | --- | --- | --- | --- | --- | --- | --- | --- | --- | --- | --- | --- | --- | --- | --- | --- |
|  | Yes  N (%) | No  N (%) | | Total  N (%) | | P | Yes  N (%) | No  N (%) | | Total  N (%) | | P | Yes  N (%) | No  N (%) | | Total  N (%) | | P |
| **Gender** |  | | | | |  |  | | | | |  |  | | | | |  |
| Female | 13 (87) | | 27 (73) | | 40 (77) | ***0.288*** | 8 (73) | | 32 (78) | | 40 (77) | ***0.701*** | 6 (67) | | 34 (79) | | 40 (77) | ***0.422*** |
| Male | 2(13) | | 10 (27) | | 12 (23) |  | 3 (27) | | 9 (22) | | 12 (23) |  | 3 (33) | | 9 (21) | | 12 (23) |  |
| **Profession** |  | |  | |  |  |  | |  | |  |  |  | |  | |  |  |
| Doctor | 4 (26,7) | | 4 (11) | | 8 (15) | ***0.076*** | 4 (36,3) | | 4 (10) | | 8 (15) | ***0.028*** | 4 (44,4) | | 4 (9) | | 8 (15) | ***0.009*** |
| Medical Technician | 4 (26,7) | | 5 (13) | | 9 (17) |  | 3 (27,3) | | 6 (15) | | 9 (17) |  | 3 (33,3) | | 6 (14) | | 9 (17) |  |
| Nurse | 6 (40) | | 13 (35) | | 19 (37) |  | 4 (36,3) | | 15 (37) | | 19 (37) |  | 2 (22,2) | | 17 (39) | | 19 (36) |  |
| Counselor | 1 (6,6) | | 15 (41) | | 16 (31) |  | 0 (0) | | 16 (38) | | 16 (31) |  | 0 (0) | | 16 (37) | | 16 (31) |  |
| **Professional Experience** |  | |  | |  |  |  | |  | |  |  |  | |  | |  |  |
| 6 M -5 years | 3 (20) | | 20 (54) | | 23 (44) | ***0.056*** | 3 (27) | | 20 (48) | | 23 (44) | ***0.426*** | 4 (44,4) | | 19 (44) | | 23 (44) | ***0.998*** |
| 6 a 10 yeras | 6 (40) | | 11 (30) | | 17 (33) |  | 5 (46) | | 12 (29) | | 17 (33) |  | 3 (33,3) | | 14 (33) | | 17 (33) |  |
| + 10 years | 6 (40) | | 6 (16) | | 12 (23) |  | 3 (27) | | 9 (23) | | 12 (23) |  | 2 (22,2) | | 10 (23) | | 12 (23) |  |
