# Supplementary material for: HIV and hepatitis B virus co-infection in Mozambique: Policy review and health professionals’ knowledge and practices
Source: PLoS One. 2024 Aug 20;19(8):e0301305. doi: 10.1371/journal.pone.0301305 (PMC11335122; doi:10.1371/journal.pone.0301305)
Supplement: S1 File — (DOCX) [file pone.0301305.s004.docx]

Questionnaire for Key Informants

**Instruction**

Explain to the participant that this interview guide will be administered to key informants who have links with the institutions involved in policy-making or who have been nominated by one of the interviewees as a potential key informant. Explain that they have been invited to take part in this research because the information they will provide us with is important for improving the provision of care for patients with HIV/Hepatitis B co-infection. Please also note that all information recorded will be kept confidential and will not be identified by name. This questionnaire should take about 30 minutes.

| **IDENTIFICATION AND SOCIODEMOGRAPHIC DATA** | | |
| --- | --- | --- |
| **1** | Date of questionnaire (dd/mm/aaaa) | \|__\|__\|- \|__\|__\|-\|__\|__\|__\|__\| |
| **2** | Participant number | \|__\|__\|-\|__\|\|__\| |
| **3** | Place |  |
| **4** | Age (years) | \|__\|__\| |
| **5** | Sex | Female\|__\| Male\|__\| |
| **6** | Profissional Category |  |
| **7** | Role of key informant |  |
| **8** | Time in Service | \|__\|__\|Months \|__\|\|__\|Years |
| **PROCESS, CONTENT, STAKEHOLDERS** | | |
| **9** | Is there currently a document regulating the management of Hepatitis B patients? | Yes\|___\|No\|___\| |
| **10** | Which type of document is it? | Policy \|___\| Strategy\|___\| Norm \|___\| Regulation\|___\| Procedure manual \|___\| Training Manual \|___\| Other\|___\| Which document?________________________________________ |
| **11** | How was the process of defining the document (timeline)? | _________________________________________________________________________________________________________________________________________________________________________________________________________________________________________________________________________________________________________________ |
| **12** | Who was involved in this process? | _______________________________________________________________________________________________________________________________________________________________________________________ |
| **MANAGEMENT OF PATIENTS WITH HBV AND HIV/HBV CO-INFECTION** | | |
| **13** | Is there currently a testing standard for the diagnosis of Hepatitis B? | Yes\|___\|No\|___\| |
| **14** | Is there a standard that dictates at what levels tests should be available? | ____________________________________________________________________________________________________________________________________________________________________________________________________________________________________________________ |
| **15** | Is there currently a rule dictating who the priority groups for Hepatitis B testing are? | Yes\|___\|No\|___\| |
| **16** | Is there any regulation dictating which professional categories can request Hepatitis B testing? | _______________________________________________________________________________________________________________________________________________________________________________________ |
| **17** | Is there any regulation that talks about Hepatitis B testing during prenatal care? | Yes\|___\|No\|___\| |
| **18** | Are there any rules dictating the referral criteria? Is there a referral flow for these patients at the district, provincial and central level? | _________________________________________________________________________________________________________________________________________________________________________________________________________________________________________________________________________ |
| **19** | Is there currently a standard for treating hepatitis B (e.g.,which patients should be treated, how they are selected, selection criteria)? | Yes\|___\|No\|___\| |
| **20** | Is there currently a standard for Hepatitis B testing in HIV-positive patients (are there specific documents)? | Yes\|___\|No\|___\| |
| **21** | Is there currently a standard for which patients should be tested or when they should be tested? | Yes\|___\|No\|___\| |
| **22** | Are there any standards for Hepatitis B follow-up and treatment for patients with co-infection? | Yes\|___\|No\|___\| |
| **23** | Are there any standards for assessing the degree of liver damage for patients with co-infection? | _______________________________________________________________________________________________________________________________________________________________________________________________________________ |
| **24** | Is there a standard for the medication available for patients diagnosed with co-infection, or which should be used? | Yes\|___\|No\|___\| |
| **25** | Is there a standard for the criteria for starting treatment? | Yes\|___\|No\|___\| |
| EXISTENCE OF HEPATITIS B VACCINES | | |
| **26** | Are there any documents that talk about the recommendations and regulations on the hepatitis B vaccine (when the vaccine started)? | Yes\|___\|No\|___\| Don't know \|___\| |
| **27** | Is there a regulation on the priority groups for the hepatitis B vaccine (is it written what the priority groups are)? | HIV positive \|__\|Pregnant Women \|__\|Health Professionals \|__\|Sex Workers \|__\| men who have sex with men \|__\| intravenous drug users \|__\| Other, which? _____________________________________________________ |
| **28** | What are the criteria for patients who want to be vaccinated (are there any rules on how they can access the vaccine)? How do you proceed in such cases? | _________________________________________________________________________________________________________________________________________________________________________________________________________________________________________________________________________ |
| **EXISTENCE OF MEDICINES FOR THE TREATMENT OF HEPATITIS B** | | |
| **29** | Are there any rules governing the import of medicines for treating Hepatitis B? | Yes\|___\|No\|___\| Don't know \|___\| |
| **30** | Is there any medicine that is imported only for Hepatitis B? | Yes\|___\|No\|___\| Don't know \|___\| |
| **31** | What kind of document is it? | Policy \|___\| Strategy\|___\| Norm \|___\| Regulation\|___\| Procedure Manual\|___\| Training Manual \|___\| Other\|___\| Which?________________________________________ |
| **EXISTENCE OF TESTS FOR HEPATITIS B** | | |
| **32** | Is there any document that talks about importing tests for Hepatitis B? | Yes\|___\|No\|___\| Don't know \|___\| |
| **33** | What kind of document is it? | Policy \|___\| Strategy\|___\| Norm \|___\| Regulation\|___\| Procedure Manual \|___\| Training Manual \|___\| Other\|___\| Which?________________________________________ |
