# Supplementary material for: HIV and hepatitis B virus co-infection in Mozambique: Policy review and health professionals’ knowledge and practices
Source: PLoS One. 2024 Aug 20;19(8):e0301305. doi: 10.1371/journal.pone.0301305 (PMC11335122; doi:10.1371/journal.pone.0301305)
Supplement: S2 File — (DOCX) [file pone.0301305.s005.docx]

Questionnaire for Health Professionals

**Instruction**

Explain to the participant that this questionnaire will be administered to health professionals who follow up and treat HIV-positive patients and aims to assess the knowledge and practices of health professionals in four health units in Maputo City.

Explain that he or she has been invited to take part in this research because the information he or she will provide us with is important for improving care for patients with HIV/Hepatitis B co-infection. Please also note that all information recorded will be kept confidential and will not be identified by name. This questionnaire should take about 45-60 minutes.

| **IDENTIFICATION AND SOCIODEMOGRAPHIC DATA** | | | |
| --- | --- | --- | --- |
| **1** | Date of questionnaire (dd/mm/aaaa) | \|__\|__\|- \|__\|__\|-\|__\|__\|__\|__\| | |
| **2** | Participant number | \|__\|__\|-\|__\|\|__\|\|__\| | |
| **3** | Health Unit |  | |
| **4** | Sex | Female\|__\| Male\|__\| | |
| **5** | Age | \|__\|__\|Years | |
| **6** | Profession | Doctor\|__\| Nurse \|__\|Medical Technician\|__\|Medical agent \|__\|Another \|__\|  Which _______________________ | |
| **7** | Time in service | \|__\|__\| years \|__\|__\|months | |
| **8** | Professional experience | \|__\|__\| years \|__\|__\|months | |
| **9** | Basic training | Basic \|__\| Middle \|__\| superior\|__\| Specialist\|__\|  Sub-specialist\|__\|Another\|__\|  Which_________________________ | |
| **10** | Specialised training | Yes \|__\|No\|__\| | |
| **11** | If yes, in which area | __________________________________________________________________________________________________________________________________ | |
| **12** | Religion | Catholic\|__\| Protestant \|__\|Muslim\|__\|Another\|__\|Which?_________________ | |
| **HIV TRAINING** | | | |
| **13** | How long have you been providing services to HIV-positive patients? | \|__\|__\| years \|__\|__\|months | |
| **14** | Have you had training in managing HIV-positive patients? | Yes \|__\|No\|__\| | |
| KNOWLEDGE ABOUT HEPATITIS B | | | |
| **15** | Have you ever heard of Hepatitis B? | Yes \|__\|No\|__\| | |
| **16** | Have you been trained to manage patients with HBV? | Yes \|__\|No\|__\| | |
| **17** | If so, when was the last training session? | \|__\|__\| years\| __\|__\|months | |
| **18** | If yes, What duration of the training? | Short duration (7 -14days) \|__\| | |
|  |  | Intermediate (15 days to 1 month) \|__\| | |
|  |  | Long duration (> 3months) \|__\| | |
| **TRANSMISSION MODES** | | | |
| **19** | Hepatitis B is transmitted by: | Sexual contact | Yes \|__\|No\|­__\|Don't know\|__\| |
|  |  | Sharing needles and syringes | Yes\|__\|No\|­__\| Don't know\|__\| |
|  |  | Blade Sharing | Yes \|__\|No\|­__\|Don't know\|__\| |
|  |  | Blood transfusion | Yes \|__\|No\|­__\|Don't know\|__\| |
|  |  | Mother to child | Yes \|__\|No\|­__\|Don't know\|__\| |
|  |  | Saliva | Yes \|__\|No\|­__\|Don't know\|__\| |
|  |  | Toothbrush sharing | Yes \|__\|No\|­__\|Don't know\|__\| |
|  |  | Tattoos, piercings, scarifications | Yes \|__\|No\|­__\|Don't know\|__\| |
|  |  | Breast milk | Yes \|__\|No\|­__\|Don't know\|__\| |
|  |  | Another  _____________________________________________________________________________________________________________________ |  |
| **MODES OF PREVENTION** | | | |
| **20** | Hepatitis B can be prevented by: | Vaccination | Yes \|__\|No\|­__\| Don't know \|__\| |
|  |  | Wearing gloves | Yes \|__\|No\|­__\| Don't know \|__\| |
|  |  | Wearing a mask | Yes \|__\|No\|­__\| Don't know \|__\| |
|  |  | Proper glove disposal | Yes \|__\|No\|­__\| Don't know \|__\| |
|  |  | Proper disposal of needles | Yes \|__\|No\|­__\| Don't know \|__\| |
|  |  | Proper disposal of syringes | Yes \|__\|No\|­__\|Don't know \|__\| |
|  |  | Use of multivitamins | Yes \|__\|No\|­__\|Don't know \|__\| |
|  |  | Safe transfusions | Yes \|__\|No\|­__\|Don't know \|__\| |
|  |  | Condom use | Yes \|__\|No\|­__\|Don't know \|__\| |
|  |  | Use of antibiotics | Yes \|__\|No\|­__\|Don't know \|__\| |
|  |  | Another  _____________________________________________________________________________________________ |  |
| **DIAGNOSIS** | | | |
| **21** | Diagnosis is made by | Hepatitis B surface antigen (HBsAg) | Yes \|__\|No\|­__\| Don't know \|__\| |
|  |  | Presence of jaundice | Yes \|__\|No\|­__\| Don't know \|__\| |
|  |  | Presence of hepatomegaly | Yes \|__\|No\|­__\| Don't know \|__\| |
|  |  | Presence of fever | Yes \|__\|No\|­__\| Don't know \|__\| |
|  |  | Urine test | Yes \|__\|No\|­__\| Don't know \|__\| |
|  |  | Blood test | Yes \|__\|No\|­__\| Don't know \|__\| |
|  |  | Another  _____________________________________________________________________________________________ |  |
| KNOWLEDGE ABOUT HIV/HEPATITIS B CO-INFECTION | | | |
| **22** | Do you know that HIV and HBV can exist in the same patient? | Yes \|__\|No\|­__\| Don't know \|__\| | |
| **23** | Have you ever heard of HBV treatment in HIV-positive patients? | Yes \|__\|No\|__\| | |
| **24** | If yes, which medications are indicated? | Tenofovir+lamivudine+Efavirenz | Yes \|__\|No\|­__\| don't know\|__\| |
|  |  | Emtricitabine+Lamivudine | Yes \|__\|No\|­__\| don't know\|__\| |
|  |  | Zidovudine+Lamivudine+Nevirapine | Yes \|__\|No\|­__\| don't know\|__\| |
|  |  | Cotrimoxazol | Yes \|__\|No\|­__\| don't know\|__\| |
|  |  | Another  ____________________________________________________________________________________________________________ |  |
| **25** | Do you know about HBV complications in patients with HIV? | Cirrhosis | Yes \|__\|No\|­__\| don't know\|__\| |
|  |  | Hepatocellular Carcinoma | Yes \|__\|No\|­__\| don't know\|__\| |
|  |  | Chronic Hepatitis | Yes \|__\|No\|­__\| don't know\|__\| |
|  |  | Seizures | Yes \|__\|No\|­__\| don't know\|__\| |
|  |  | Hepatotoxicity to Antiretrovirals | Yes \|__\|No\|­__\| don't know\|__\| |
|  |  | Another  ______________________________________________________________________________________________________ |  |
| **PRACTICES REGARDING THE MANAGEMENT OF HIV/HEPATITIS B CO-INFECTION** | | | |
| **26** | Do you screen for HBV in a patient with HIV? | Yes\|__\|No\|__\| | |
| **27** | Do you counsel to prevent HBV transmission? | Yes \|__\|No\|__\| | |
| **28** | Do you counsel on risky behavior? | Yes\|__\|No\|__\| | |
| **29** | If so, what risky behaviors? | Alcohol consumption Yes\|__\|No\|­__\|Don't know\|__\| | |
|  |  | Use of traditional medicine Yes\|__\|No\|__\|Don't know\|__\| | |
|  |  | Intravenous drug use Yes\|__\|No\|­__\|Don't know\|__\| | |
|  |  | Sharing of syringes and needles Yes\|__\|No\|­__\|Don't know\|__\| | |
|  |  | Condom use Yes\|__\|No\|__\|Don't know\|__\| | |
|  |  | Another  _____________________________  _____________________________  _____________________________ | |
| **30** | What signs do you look for in the patient with co-infection? | Jaundice Yes\|__\|No\|__\|Don't know\|__\| | |
|  |  | Hepatomegaly Yes\|__\|No\|__\|Don't know\|__\| | |
|  |  | Ascites Yes\|__\|No\|__\|Don't know\|__\| | |
|  |  | Splenomegaly Yes\|__\|No\|__\|Don't know \|__\| | |
|  |  | Changes in the skin Yes\|__\|No\|__\|Don't know\|__\| | |
|  |  | Another  _____________________________  _____________________________  _____________________________ | |
| **31** | Have you ever treated/followed a patient with Hepatitis B? | Yes\|__\|No\|__\| | |
| **32** | How many patients do you have with hepatitis B? | \|__\|__\|__\| | |
| **VACCINATION STATUS** | | | |
| **33** | Have you ever been vaccinated against Hepatitis B virus? | Yes\|__\|No\|__\| don't know\|__\| | |
| **34** | Do you know when you were vaccinated*  (those who reported having had at least 1 dose of the vaccine) | Yes\|__\|No\|__\|don't know\|__\| | |
| **35** | If yes, say when was the last dose | < 6 months  6 months – 1 year  > 1 year | |
| **36** | How many doses did you get? (those who reported having the vaccine) | 1 dose  2 doses  3 doses | |
| **37** | Do you think the number of doses you received was correct? | Yes\|__\|No\|__\|Don't know\|__\| | |
| **38** | If not, say how many you should receive | 2 doses  3 doses | |

HIV- human immunodeficiency virus, HBV-hepatitis B virus
